# Solid Lipid Nanoparticles Based on Babassu Oil and Copaiba Oleoresin: A Promising Approach for Prostate Cancer Therapy

**DOI:** 10.3390/nano14121014

**Published:** 2024-06-12

**Authors:** Michael Jackson Ferreira da Silva, Alisson Mendes Rodrigues, Maria Célia Pires Costa, Adriana Leandro Camara, Lucio Mendes Cabral, Eduardo Ricci Junior, Daniel Figueiredo Vanzan, Ana Paula dos Santos Matos, Thiago da Silva Honorio, Antonio Carlos Romão Borges

**Affiliations:** 1 Programa de Pós-Graduação em Biotecnologia da Rede Renorbio, Universidade Federal do Maranhão (UFMA), Av. dos Portugueses, 1966, Bacanga, São Luís 65080-805, MA, Brazil; michael_qmc@hotmail.com (M.J.F.d.S.);; 2 Programa de Pós-Graduação em Ciência de Materiais, Faculdade UnB Planaltina, Universidade de Brasília (UnB), Brasília 70904-910, DF, Brazil; 3 Departamento de Química, Universidade Estadual do Maranhão (UEMA), Campus Universitário Paulo VI, São Luís 65055-970, MA, Brazil; celiacosta@prof.elointernet.com.br; 4 Departamento de Ciências Fisiológicas, Universidade Federal do Maranhão (UFMA), Av. dos Portugueses, 1966, Bacanga, São Luís 65080-805, MA, Brazil; al.camara@ufma.br; 5 Departamento de Fármacos e Medicamentos, Faculdade de Farmácia, Universidade Federal do Rio de Janeiro (UFRJ), Cidade Universitária, Ilha do Fundão, Rio de Janeiro 21941-902, RJ, Brazil; lmcabral@pharma.ufrj.br (L.M.C.); ricci@pharma.ufrj.br (E.R.J.); anapaulasmatos@gmail.com (A.P.d.S.M.);

**Keywords:** solid lipid nanoparticles, babassu oil, copaiba oleoresin, PC-3 and DU-145 prostate cancer lines

## Abstract

Solid lipid nanoparticles (SLNs) represent promising nanostructures for drug delivery systems. This study successfully synthesized SLNs containing different proportions of babassu oil (BBS) and copaiba oleoresin (COPA) via the emulsification–ultrasonication method. Before SLN synthesis, the identification and quantification of methyl esters, such as lauric acid and β-caryophyllene, were performed via GC-MS analysis. These methyl esters were used as chemical markers and assisted in encapsulation efficiency experiments. A 2^2^ factorial design with a center point was employed to assess the impact of stearic acid and Tween 80 on particle hydrodynamic diameter (HD) and polydispersity index (PDI). Additionally, the effects of temperature (8 ± 0.5 °C and 25 ± 1.0 °C) and time (0, 7, 15, 30, 40, and 60 days) on HD and PDI values were investigated. Zeta potential (ZP) measurements were utilized to evaluate nanoparticle stability, while transmission electron microscopy provided insights into the morphology and nanometric dimensions of the SLNs. The in vitro cytotoxic activity of the SLNs (10 µg/mL, 30 µg/mL, 40 µg/mL, and 80 µg/mL) was evaluated using the MTT assay with PC-3 and DU-145 prostate cancer cell lines. Results demonstrated that SLNs containing BBS and COPA in a 1:1 ratio exhibited a promising cytotoxic effect against prostate cancer cells, with a percentage of viable cells of 68.5% for PC-3 at a concentration of 30 µg/mL and 48% for DU-145 at a concentration of 80 µg/mL. These findings underscore the potential therapeutic applications of SLNs loaded with BBS and COPA for prostate cancer treatment.

## 1. Introduction

Solid lipid nanoparticles (SLNs) have arisen as a pioneering strategy for crafting novel nanocarriers, diverging from liposomes, which have traditionally been regarded as conventional models [1,2]. SLNs have aroused significant interest due to their diverse properties, including the ability to traverse biological barriers, variability in particle size, and an attractive cost–benefit ratio, positioning them as a highly viable lipid delivery system [3]. Comprising a solid lipophilic core, these nanoparticles can incorporate drugs and other molecules, especially lipophilic ones, between the fatty acid chains of their lipid matrix [1]. The capacity to target SLN to tumor regions through exploiting the physical and chemical differences and biological interactions between tumor and normal cells emerges as a promising strategy [4]. Furthermore, their unique characteristics, such as ease of manipulation, biocompatibility, and a large surface area, make these systems effective carriers in technologies aimed at cancer therapy [5,6].

SLNs represent a highly attractive delivery system for the pharmaceutical industry, capable of incorporating lipophilic substances like oils with low solubility in water, limiting their bioavailability and therapeutic potential [3]. Among the most used techniques to produce lipid nanoparticles, the following stand out: solvent evaporation, supercritical fluid extraction from emulsions, multiple emulsions, spray homogenization, ultrasound, high-speed homogenization, and ultrasound [7]. Lately, the emulsion ultrasound method, a variation of the ultrasound method [8,9,10,11], has been widely used for controlling the hydrodynamic diameter (HD) and polydispersity index (PDI) of SLNs.

HD determination is crucial during SLN preparation, preventing splenic retention [12]. Reducing HD is important, as SLN passes through the fenestrae of neoplastic tissues with a diameter of 300 at 900 nm [13,14]. A PDI below 0.4, equal to or less than 0.3, is recommended for the therapeutic efficacy of cancer-targeted nanoparticles [15]. Additionally, SLNs with spherical morphologies are advantageous due to greater mobility resulting from their symmetry. In contrast, non-spherical ones are more prone to sedimentation, especially during filtration in organs like the spleen and liver [16]. Thus, the spherical nanosystem morphology plays a crucial role in blood circulation and cellular interactions compared with non-spherical counterparts [17].

Prostate cancer is the second most common malignancy among men globally, surpassed only by non-melanoma skin cancer [18,19]. The incidence of prostate cancer is significant, with over 1.4 million novel cases estimated in 2020 and more than 375,000 deaths related to the disease in the same year [20]. Projections continue to increase, with an estimated 2.43 million novel cases in 2040 [21,22,23]. Androgen deprivation therapy, aiming to suppress androgen receptor signaling, remains the preferred method in prostate cancer treatment [24,25]. However, despite the initial positive response, prolonged androgen receptor suppression often leads to prostate cancer resistance, progressing to the castration-resistant prostate cancer stage, also known as androgen-independent prostate cancer [26,27].

Notably, no study in the literature has used SLN with a combination of babassu (BBS) oil and copaiba (COPA) oleoresin for prostate cancer treatment [28,29,30]. BBS oil (*Attalea speciosa*) comprises various fatty acids such as lauric acid (40–55%), myristic acid (11–27%), oleic acid (10–16%), stearic acid (1.8–7.4%), palmitic (5–11%), capric (1.2–8%), and caprylic (6%) acids [31,32]. Lauric acid is particularly noteworthy for its therapeutic properties, including antibacterial, anti-inflammatory, and antitumor activities [33]. COPA oleoresin (*Copaifera multijuga* Hayne) consists of a non-volatile or sesquiterpene part, accounting for 10–15% or up to 80% of vegetable oil [34], with emphasis on the compounds β-caryophyllene, β-bisabolene, α-humulene, α and β-selinene, α-bisabolol, β-element, γ-cadinene, and α-cadinol [35]. The less volatile, resinous, or diterpenic part includes kaurenoic, polylactic, and copalic acids [34]. The pharmacological potential of COPA oleoresin is mainly due to its anti-inflammatory [36], antibacterial [37], antifungal [38], and antitumor properties [39].

In this study, SLNs containing BBS oil and COPA oleoresin were developed as novel carriers for prostate cancer therapy. These SLNs were produced using the emulsification-ultrasonication method. The impact of the lipid matrix, nonionic surfactant, temperature, and time on the HD and PDI was systematically evaluated. Cell viability was determined by MTT assay using PC-3 and DU-145 human prostate cancer cell lines. Our findings demonstrated promising results in the efficiency of the SLN preparation method and reduction of cell viability in prostate cancer cell lines using SLN-BBS-COPA nanoparticles. The employed SLN preparation method may be viable and scalable for industrial production. These findings have the potential to propel the development of novel treatment strategies for prostate cancer, the second most prevalent cancer in men. This approach holds significant promise in addressing the escalating demand for effective and innovative therapeutic alternatives for prostate cancer, marking a substantial advancement in oncology. Beyond their therapeutic potential for prostate cancer, BSS oil and COPA oleoresin are natural and abundant species in Brazil’s northeast and northern regions. This innovative application adds value to these resources and contributes to regional development.

## 2. Materials and Methods

**Figure 1:**
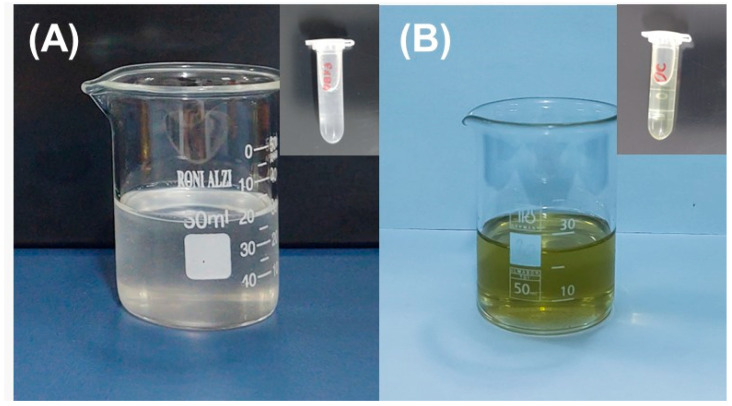
(**A**) babassu oil and (**B**) copaiba oleoresin.

The babassu oil (BBS) was extracted using the cold pressing technique. Babassu coconut almonds were purchased from coconut breakers (Cooperquilombola) in Penalva City, Maranhão State, Brazil. A representative botanical specimen was cataloged and deposited in the Ático Seabra Herbarium at the Federal University of Maranhão (Brazil) with the identification number 01371. Copaiba oleoresin (COPA) was sourced at the local market in the Alto Trombetas Oriximiná district, Pará State, Brazil. Other reagents used were polyoxyethylene sorbitan monooleate 80 (Tween 80), used as a nonionic surfactant and acquired from Tedia (Rua Sousa Barros, Brazil), and stearic acid (<90%, Êxodo Científica, Sumaré, Sao Paulo, Brazil), which was used as the lipid matrix. Figure 1 illustrates the vegetable oils utilized for the SLN preparation.

### 2.1. Gas Chromatography Coupled to Mass Spectrometer (CG-MS)

The GC-MS experiments were performed in a gas chromatograph coupled to a mass spectrometer (QP2010 Ultra model, Shimadzu, Barueri, Brazil), equipped with a quadrupole detector and a capillary column composed of 5% diphenyl–95% dimethyl polysiloxane (RXi—5 ms; 30 m × 0.25 mm × 0.25 μm Df; Restek, Bellefonte, PA, USA). BBS triacylglycerols were hydrolyzed and methylated before injection. COPA was derivatized with dichloromethane and then injected. He(g) was used as the carrier gas at a 1.8 mL/min flow rate. The temperature program was set as follows: 30 °C, held for 1 min; 30 °C to 150 °C (20 °C/min), held for 3 min; and 150 °C to 310 °C (25 °C/min), held for 5 min. Samples were dissolved in dichloromethane (5 mg/mL), and 1 µL of the sample was injected with splitless flow at a ratio of 1:200. The ion source temperature was maintained at 230 °C. The quadrupole detector was operated in electron ionization mode with a mass range collected from 30–300 *m*/*z*. Methyl pentadecanoate (Sigma Aldrich, St. Louis, MO, USA) was used as an internal standard. Lauric acid (Sigma Aldrich, USA) was employed as a chemical marker for BBS, while β-caryophyllene (Sigma Aldrich, USA) was utilized for COPA.

### 2.2. Experimental Design

A 2^2^ factorial experimental design with a center point was used to assess the effects of the independent variables (stearic acid (%) and Tween 80 (%)) on the particle hydrodynamic diameter (HD) and polydispersity index (PDI). The HD and PDI values were measured immediately after SLNs preparation (t_0_). The variance analysis (ANOVA) was employed to ascertain the statistical significance of the independent variables concerning HD and PDI, with a 95% confidence level. Linear and quadratic mathematical models were adjusted to the experimental data to evaluate which model was more representative and predictive. All experiments were performed in triplicate. The 2^2^ factorial experimental design, analysis of variance, and mathematical model adjustments were performed using Statistica 10 software. Table 1 presents the decoded values of the independent variables.

### 2.3. Preparation of Solid Lipid Nanoparticles (SLNs)

**Figure 2:**
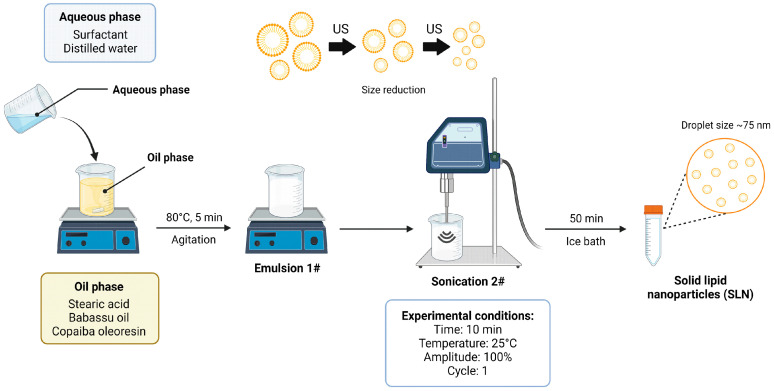
Schematic of the experimental procedure used to prepare the SLNs studied in this work.

The SLNs were prepared using the modified emulsification–ultrasonication method. In summary, SLNs with different BBS and COPA proportions were prepared through combining the oil and aqueous phases. For this purpose, 4 g of stearic acid was melted at 70 °C to obtain the oil phase. Concomitantly, 2.5 g of Tween 80 was dissolved in 100 mL of distilled water. Then, both the aqueous and oily phases were heated to 80 °C, but only the aqueous phase was subjected to constant stirring. Before combining the aqueous and oil phases, BBS (1 g) and COPA (1 g) were dissolved in the oil phase. The aqueous and oil phase mixture was accomplished under magnetic stirring at 80 °C for 5 min, followed by sonication in an ultrasonic processor (UP100H, Hielscher Ultrasonics, Teltow, Germany) operating at 100 W and 30 kHz, with 100% amplitude and cycle 1, for 10 min. Then, the mixture was cooled in an ice bath for 50 min. The SLN-BBS, SLN-COPA, and SLN-W were prepared using the same experimental procedure used for SLN-BBS-COPA, with modifications tailored to BBS and COPA-specified proportions; see Table 2. The SLNs were investigated in aqueous solution form. Figure 2 shows the main steps used in the SLNs’ preparation.

### 2.4. Characterization of Solid Lipid Nanoparticles

HD and PDI values were measured through dynamic light scattering (Zetasizer Nano^®^ S90 model, Malvern, Westborough, MA, USA). The SLNs were diluted in distilled water at a 1:100 ratio, and the measurements were taken at different times (t = 0, 7, 15, 30, 40, and 60 days) at different temperatures (RT = 25 ± 1.0 °C and UR = 8 ± 0.5 °C, refrigeration condition). Table 3 outlines the nomenclature utilized for the SLNs during the stability study.

Zeta potential measurements (ζ) of SLNs were performed in a Zeta Potential Analyzer (NanoBrook ZetaPALS model, Brookhaven, Holtsville, NY, USA). The samples were diluted in distilled water in a ratio of 1:16 (*v*/*v*), and 1.5 mL specimens of the solutions placed in polystyrene cuvettes with four polished sides. Analyzes were performed in triplicate at room temperature.

### 2.5. Morphological Analysis by Transmission Electron Microscope (TEM)

The morphological characteristics of the SLNs were evaluated using a transmission electron microscope (HT 7800 model, Hitachi, Minato-ku, Tokyo) at 80 kV. To prepare the samples, 330 µL of the SLN formulation was deposited on a copper grid (50 μm) covered with Formvar^®^. To obtain contrast, 10 µL of a 5% (*w*/*v*) uranyl acetate solution was added, and the excess solution was removed with filter paper for subsequent observation under the microscope. 

### 2.6. Oil Encapsulation Efficiency

The encapsulation efficiency (EE) of BBS and COPA in SLNs was evaluated according to the following experimental procedure: known quantities of SLNs containing different proportions of BBS and COPA were diluted in methanol to disrupt their structure and expose the natural bioactives. Then, methanol was removed via evaporation, followed by derivatization of BBS and COPA as described by [40]. The resulting mixture was diluted in ethyl acetate, and methyl pentadecanoate was used as an internal standard [41]. Lauric acid and β-caryophyllene were used as chemical markers of BBS and COPA, respectively, and were quantified via GC-MS. For this, calibration curves were prepared from mixtures of marker standards in which a stock solution with a concentration of 4509 μg/mL was systematically diluted to obtain varied concentrations (50, 150, 200, 300, 400, 500 and 650 μg/mL), all in triplicate.

The samples were dissolved in dichloromethane (5 mg/mL), and a small amount was injected into the GC-MS with flow division at a ratio of 1:200 (splitless). Based on the peak areas obtained from the mentioned concentrations, calibration curves were developed for the two markers, resulting in linear equations (Equations (1) and (2)):

Y1 = 0.0011x + 0.0061 onde R^2^ = 0.9919 (1)

Y2 = 0.00003x + 0.0007 onde R^2^ = 0.9947 (2)

where Equations (1) and (2) represent the concentrations of lauric acid and β-caryophyllene.

The EE values of BBS and COPA oils in SLNs were calculated via Equation (3):

EE = [CDM/CTM] × 100(3)

where CDM and CTM correspond to the determined concentration of the marker and the theoretical concentration of the marker in the SLN, respectively.

### 2.7. In Vitro Cytotoxicity

#### 2.7.1. Cell Lines

The PC-3 and DU-145 human prostate cancer cell lines were acquired from the Rio de Janeiro Cell Bank (BCRJ, Rio de Janeiro, Brazil). PC-3 cells were maintained in Roswell Park Memorial Institute (RPMI) medium supplemented with 10% (*v*/*v*) fetal bovine serum (FBS). DU-145 cells were cultured in Dulbecco’s Modified Eagle Medium (DMEM), supplemented with 10% FBS, 1% streptomycin, and amphotericin. Cultures were incubated in humidified conditions with an atmosphere of 95% air and 5% CO_2_. The choice of lines was based on their representation of the main aspects of androgen-independent human prostate cancer and its progression [42].

#### 2.7.2. Cell Viability MTT Assay

The SLNs’ cytotoxicity was assessed via the 3-(4,5-dimethylthiazol-2-yl)-2,5-diphenyltetrazolium bromide (MTT) assay [43]. To achieve this, 200 μL of PC-3 or DU-145 culture cells medium was put into a 96-well plate, 5 × 10^4^ cells per well. The plate was then incubated with 95% air and 5% CO_2_. Then, monolayer cells were exposed to 10, 30, 40, and 80 μg/mL of SLN-BBS-COPA, SLN-BBS, SLN-COPA, SLN-W, BBS, COPA, and BBS + COPA. Flutamide (FTM) was used as an antineoplastic control [42]. It was prepared from a dimethyl sulfoxide (DMSO) PA solution and diluted to a concentration of 25 μM with 1% DMSO. After 24 h of incubation, additional MTT in phosphate-buffered saline (PBS) (0.5 mg/mL) was added under pH = 7.4, followed by a 3 h incubation. Reading at 570 nm, after solubilization of the formazan crystals with DMSO, revealed the percentage of cell viability.

### 2.8. Statistical Analysis

Experimental data were expressed as mean ± standard deviation (SD). For comparison between groups, one-way and two-way ANOVA analyses of variance were performed, followed by the post-Tukey test for evaluations involving three or more experimental groups. Values of *p* < 0.05, *p* < 0.01, *p* < 0.001, and *p* < 0.0001 were considered statistically significant and were represented in the figures by *, **, ***, and ****, respectively. All statistical analyses were conducted using GraphPad Prism^®^ 9 software (GraphPad Software, Version 9.0.0, San Diego, CA, USA).

## 3. Results and Discussion

### 3.1. Identification of Methyl Esters of Natural Bioactives (BBS and COPA) via GC-MS

**Figure 3:**
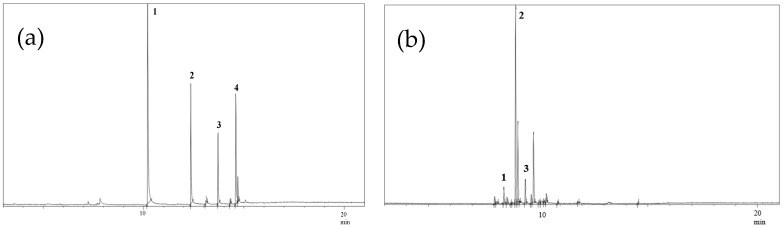
GC-MS spectra obtained from (**a**) BBS and (**b**) COPA.

The quantitative analysis of methyl esters present in natural bioactives (BBS and COPA) was performed using the GC-MS technique; see Figure 3a,b. In the GC-MS spectrum of BBS (Figure 3a), the most prominent peak (peak 1) was correlated to lauric acid, which was selected as a chemical marker for this natural bioactive. In addition to lauric acid, other acids such as myristic, palmitic, and oleic acids were also identified. The mass spectra of lauric acid exhibited three high-intensity ions, *m*/*z* 55, *m*/*z* 74, and *m*/*z* 87, which were used for integration during the quantification of BBS content. In the case of COPA (Figure 3b), the most significant peak corresponded to β-caryophyllene (peak 2), establishing itself as an identifiable chemical marker for COPA. These results corroborate previous studies, as described by Abreu et al. [40]. Detailed information about the methyl esters identified in the BBS and COPA is shown in Table 4 and Table 5, respectively. In these tables, the analytes are listed along with their retention times and the corresponding *m*/*z* values, which were the characteristic ions used to identify and quantify these compounds.

### 3.2. Preparation and Characterization of SLNs

#### 3.2.1. Influence of the Lipid Matrix and Nonionic Surfactant on HD and PDI Values

The 2^2^ factorial experimental design with a center point was applied to assess the effect of the lipid matrix (stearic acid) and nonionic surfactant (Tween 80) on the HD and PDI values of SLNs containing BBS and COPA. For this purpose, HD and PDI values were measured from SLN compositions recommended according to the initial analysis conducted as part of the experimental design; see Table 6. It was observed that an increase in the stearic acid concentration resulted in higher HD values, as observed in SLN1 and SLN2, which measured 242.8 ± 3.20 nm and 389.2 ± 64.70 nm, respectively. Conversely, an increase in the Tween 80 concentration led to a decrease in HD values. For example, SLN 1 (without Tween 80) presented a larger HD value than SLN 3 (98.0 ± 1.20), to which 5% Tween 80 had been added. The same behavior was observed for SLN 2 and SLN 4. It is known that increasing the surfactant concentration reduces the surface tension at the interface of the aqueous and oil phases. This contributes to the homogenization of the system and, therefore, decreases the SLN sizes [44]. Unlike what was observed for the HD values, the increase in stearic acid concentration appears to have had a non-linear relationship with PDI. For example, comparing SLN 1 and SLN 2, both with Tween 80 concentration equal to 0%, the PDI increased significantly from 0.168 ± 0.010 to 0.522 ± 0.139 when the stearic acid concentration was raised from 2% to 6%, respectively. On the other hand, increasing the stearic acid concentration from 2% (SLN 3) to 6% (SLN 4) decreased PDI from 0.393 ± 0.026 to 0.285 ± 0.008. Based on these results, the addition of 5% Tween 80 influenced PDI values, promoting a more homogeneous distribution in particle size. However, in subsequent analyses, this SLN showed signs of destabilization.

The linear and quadratic mathematical models were adjusted to the experimental data shown in Table 7. The adjustment results are shown in Table 4. The most representative and predictive adjustments were chosen based on three criteria: (i) presenting a *p*-value lower than 0.05, (ii) presenting Ftest greater than 5 (Ftest > 5), and (iii) presenting a higher R^2^ value. Therefore, based on these criteria, the quadratic and linear models were considered the most representative and predictive for HD and PDI values, respectively. Equations (4) and (5) represent the dependence of HD and PDI values as a function of the stearic acid and Tween 80 amounts. The x and y coefficients correspond to the stearic acid and Tween 80 concentrations in these equations, respectively. All coefficients were statistically significant at the 95% confidence level.

(4) $$\mathrm{HD}\;\left(x,y\right)=15.075+139.617*x\;-\;12.877\;*\;x^{2}-\;30.49*y+0.765*x*y+0$$

(5) $$\mathrm{PDI}\;\left(x,y\right)=0.243\;-\;0.0795*x+0.021\;*\;x^{2}+0.091*y\;-\;0.0231*x*y\;+\;0$$

**Figure 4:**
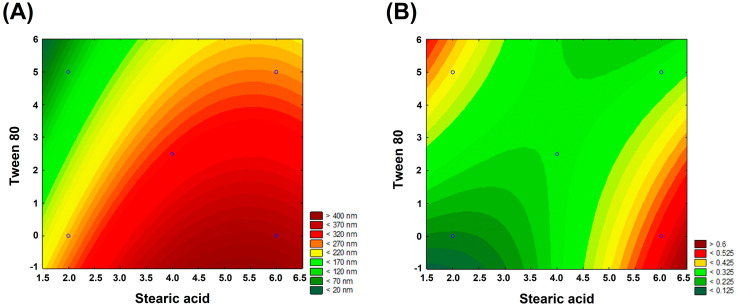
2D response surface graphs obtained from the experimental design applied to the (**A**) HD and (**B**) PDI.

Figure 4A,B shows the 2D response surface graphs obtained from Equations (1) and (2). Figure 4A shows the highest HD values (>400 nm) obtained from the SLNs containing higher concentrations of stearic acid and Tween 80 concentrations equal to or approaching zero. On the other hand, the lowest PDI values (<0.125) were observed for SLNs with low concentrations of stearic acid (<3.5%) and Tween 80 concentrations equal to or approaching zero (Figure 3b). These observations align with the experimental data presented in Table 6.

#### 3.2.2. Time and Temperature Dependence of the HD Values

**Figure 5:**
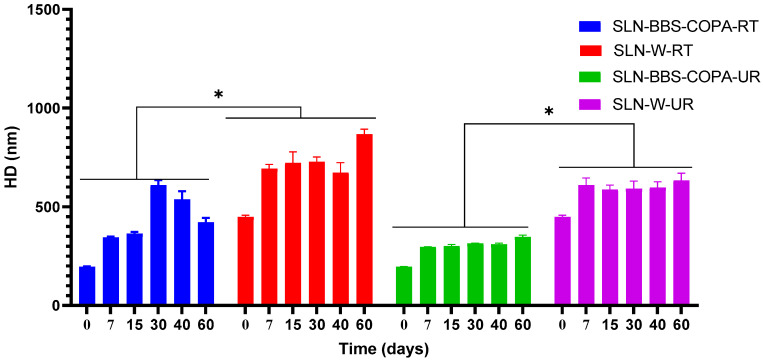
Effect of time and temperature on HD values of SLNs with (SLN-BBS-COPA-RT, SLN-BBS-COPA-UR) and without (SLN-W-RT and SLN-W-UR) natural bioactives.

HD experimental data of the SLN-BBS-COPA and SLN-W samples measured at different times (t = 0, 7, 15, 30, 40, and 60 days) and temperature conditions (UR = 8 °C and RT = 25 °C); see Figure 5. In general, HD values at t = 0 days were lower for the same sample than those measured at longer times (t = 7, 15, 30, 40, and 60 days). HD values were also sensitive to temperature since those values measured from SLN-BBS-COPA held at 8 °C were significantly lower (*p* < 0.05) than those measured from SLN-BBS-COPA held at 25 °C. Furthermore, a smaller variation in HD values was observed for samples kept at 8 °C for 7 to 60 days. This behavior indicates that these formulations (SNL-BBS-COPA-UR and SLN-W-R) were stable under refrigeration [45,46]. It was also possible to verify the influence of natural bioactives (BBS and COPA) on HD values. Generally, SLNs containing BBS and COPA presented lower HD values than their respective SLNs without these components (SLN-W-UR and SLN-W-RT, respectively).

It is important to highlight that HD values greater than 400 nm are not recommended for SLNs aimed at cancer treatment, since the fenestrae in the tumor vasculature range from 300 to 900 nm [13]. The HD values measured from the SLN-BBS-COPA-RT sample were lower than 400 nm (345.5 ± 3.38 nm and 364.60 ± 8.32 nm to t = 7 and 15 days, respectively) and increased for samples kept at t = 30 days (609.07 ± 26.40 nm), t = 40 days (537.90 ± 40.84 nm), and t = 60 days (421.87 ± 22.30 nm). Such an increase in HD values from t = 30 days can be attributed to the recrystallization of the lipid phase, which can contribute to the decrease in the long-term stability of SLN aqueous dispersions [3].

#### 3.2.3. Time and Temperature Dependence of the PDI Values

**Figure 6:**
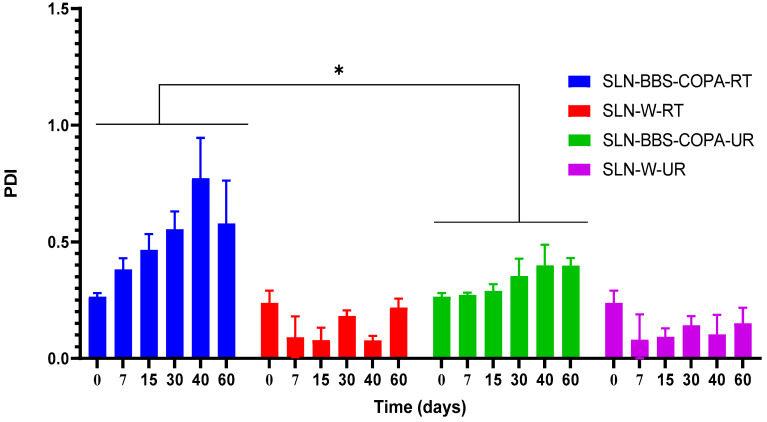
Effect of time and temperature on PDI values of SLN with (SLN-BBS-COPA-RT, SLN-BBS-COPA-UR) and without natural bioactives (SLN-W-RT and SLN-W-UR).

The PDI values of SLN-BBS-COPA and SLN-W samples were also evaluated at different times (0, 7, 15, 30, 40, and 60 days) and temperatures (UR = ±8 °C and RT = ±25 °C); see Figure 6. For SLN-BBS-COPA-UR, the PDI values ranged from 0.265 ± 0.015 at t = 0 days and 0.398 ± 0.033 at t = 60 days, but without a statistical difference (*p* > 0.05). SLN-BBS-COPA-RT presented a significant increase (*p* < 0.05) in PDI values at t = 15 days onwards, being characteristic of a polydisperse system (PDI > 0.4). It was possible to observe a statistically significant difference (*p* < 0.05) in the PDI values of the SLN-BBS-COPA-UR and SLN-BBS-COPA-RT groups over time. It is known that an acceptable PDI value should be equal to or less than 0.3, as this guarantees a distribution of SLN particles [15]. On the other hand, SLN-W-RT and SLN-W-UR presented a range of PDI values lower than < 0.1. However, these results did not correlate with the HD values measured for SLN-W-RT and SLN-W-UR, especially for SLN-W-RT with an HD greater than 400 nm. The higher HD values of SLN-W-RT and SLN-W-UR were probably influenced by the equipment signal, resulting in an overestimation of the average HD and, consequently, a decrease in the PDI values [47,48]. The correlation between HD and PDI values (equal to or less than 0.3) is relevant for pharmaceutical strategies aimed at cancer treatment, which exploit the effects of increased permeability and retention in neovascularized solid tumor tissues [49,50].

#### 3.2.4. Zeta Potential (ξ) Measurements

**Figure 7:**
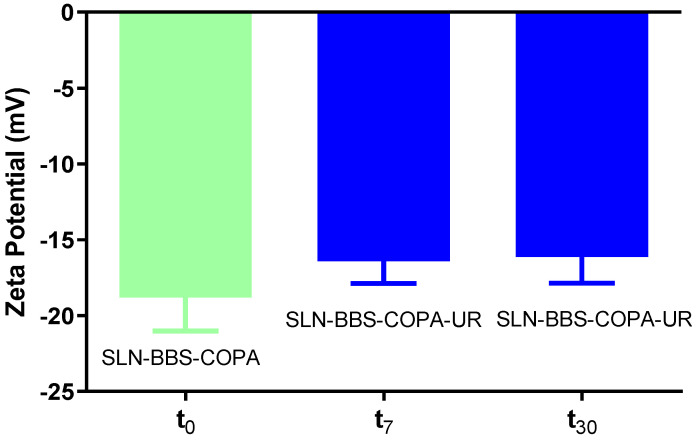
ξ measurements of the SLN-BBS-COPA-UR at different times (0, 7, and 30 days).

The ξ measurements were performed only on SLN-BBS-COPA-UR samples kept for 0, 7, and 30 days, see Figure 7. These samples were chosen because their HD and PDI values were lower to 400 nm and 0.4, respectively, which is recommended for treating tumor cells. It is known that the ξ measurements characterize the charge on the nanoparticles’ surface; therefore, they can be used to predict the long-term stability of a dispersion [45,51]. At times t = 0, 7, and 30 days, the ξ values for SLN-BBS-COPA-UR were −18.82 ± 1.96 mV, −16.40 ± 1.49 mV, and −16.14 ± 1.72 mV, respectively. The negative charge measured in the system correlated with the dissociation of stearic acid in the aqueous medium [52]. In general, the ξ values measured were negative for all times investigated and suggested that the surface electrical charge was not a dominant factor in the physical stability of the SLN-BBS-COPA-UR. On the contrary, the results indicate that the critical factor for maintaining stability lies in the steric hindrance associated with the nonionic surfactant that covers the SLN [53]. This reinforces the hypothesis that the stability of SLN-BBS-COPA-UR is intrinsically associated with the steric interactions promoted by the surfactant coating. The conventional boundary between stable and unstable suspensions is usually set at +30 or −30 mV, classifying electrically charged particles in these ranges as stable [54].

### 3.3. Morphology

**Figure 8:**
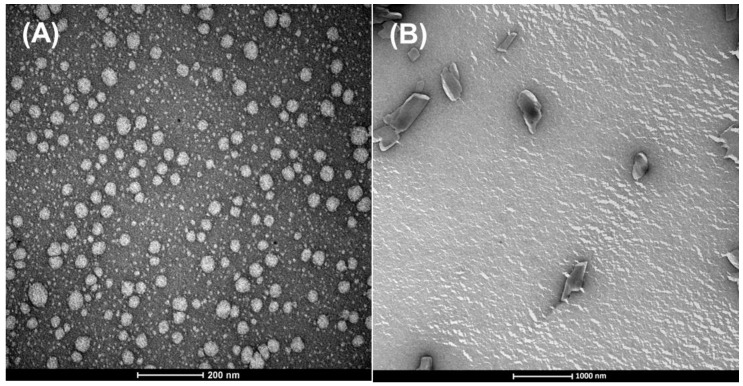
TEM images acquired from (**A**) SLN-BBS-COPA and (**B**) SLN-W samples.

The morphology of SLN-BBS-COPA and SLN-W samples was evaluated via TEM analysis (Figure 8). As expected, it was confirmed that both SLNs presented nanometric dimensions (Figure 8A,B). However, the SLN-BBS-COPA average size (~75 nm) was six times smaller than SLN-W (~500 nm). Also, SLN-BBS-COPA presented a spherical shape, while SLN-W presented a non-spherical morphology (oval or rod). Regardless of the different administration routes, it is important to highlight that the SLNs’ morphology plays a significant role in transport and diffusion. It is already known that spherical particles have greater ease of movement due to their symmetry, while non-spherical particles tend to follow the flow differently, particularly in organs such as the spleen and liver during filtration [16]. No signs of crystallization were observed in SLN-BBS-COPA (Figure 8A). Crystals on the surface of the nanoparticles could be related to the lipid’s crystalline characteristics.

### 3.4. Encapsulation Efficiency (EE) of BBS and COPA

**Figure 9:**
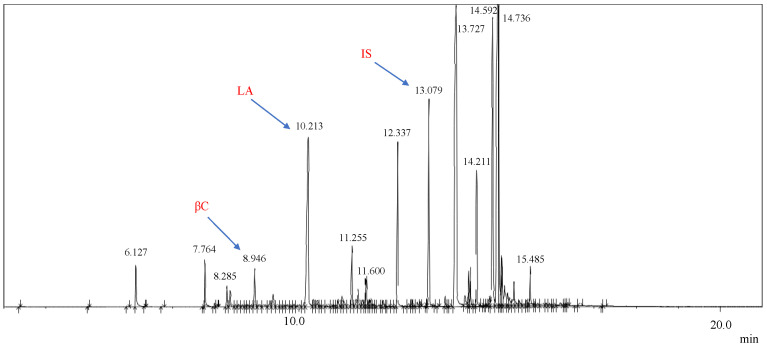
GC-MS spectrum of SLN-BBS-COPA.

The BBS and COPA encapsulation efficiency in SLN-BBS-COPA was evaluated via GC-MS measurements [55]. Figure 9 shows the GC-MS spectrum of SLN-BBS-COPA, in which it is possible to identify distinct peaks corresponding to lauric acid (LA), β-caryophyllene (βC), and the internal standard (methyl pentadecanoate). The LA and βC quantifications were performed using calibration curves (Equations (1) and (2)), while encapsulation efficiency values were determined using Equation (3). The results showed that the BBS and COPA encapsulation efficiencies in the SLN-BBS-COPA were equal to 69.96% and 92.87%, respectively. These findings indicate the remarkable ability of SLNs to incorporate BBS and COPA efficiently into their lipid matrix. The lipophilic nature of these compounds favors their miscibility with the lipid matrix, resulting in significant retention. Furthermore, lipid-based delivery systems offer protection against chemical, photochemical, or oxidative degradation of sensitive oil components due to their encapsulation within solid lipids, as discussed in reference [56].

### 3.5. In Vitro Cytotoxicity Assessment

**Figure 10:**
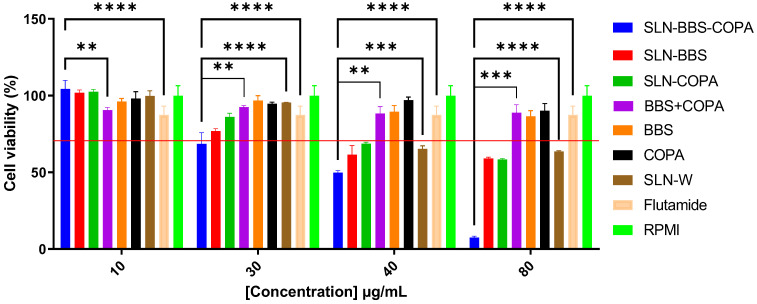
Cell viability of the PC-3 line in different concentrations of SLN-BBS-COPA, SLN-BBS, SLN-COPA, SLN-W, BBS, COPA, BBS + COPA, and free drug (flutamide) after 24 h of exposure. The red line indicates 70% cell viability according to ISO:10993-5 [57].

**Figure 11:**
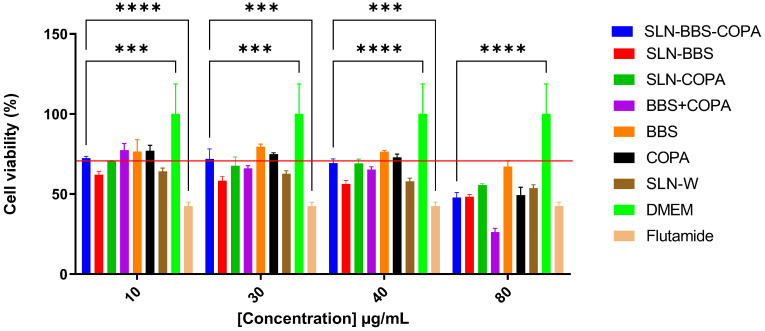
Cell viability of the DU-145 line in different concentrations of SLN-BBS-COPA, SLN-BBS, SLN-COPA, SLN-W, BBS, COPA, BBS + COPA and free drug (flutamide) after 24 h of exposure. The red line indicates 70% cell viability according to ISO:10993-5 [57].

Figure 10 and Figure 11 present the cell viability of PC-3 and DU-145 cell lines when separately exposed to SNLs containing different proportions of BBS and COPA (1:1, 1:0, 0:1, and 0:0), as well as to isolated natural bioactives (BBS + COPA, BBS, and COPA), flutamide (positive control), RPMI (negative control), and DMEM (negative control). Table 8 shows the concentration of the SLNs (10 µg/mL, 30 µg/mL, 40 µg/mL, and 80 µg/mL) used to perform the in vitro cytotoxicity tests and the BBS and COPA quantities used to obtain these concentrations.

The influence of SLN concentration on PC-3 cell viability is shown in Figure 10. SLN-BBS-COPA showed a viable cell rate of 68.5% at 30 µg/mL, while SLN-BBS, SLN-COPA, SLN-W, BBS, COPA, and BBS + COPA showed viable cell rates of 76.9%, 86.1%, 95.4%, 96.7%, 94.7%, and 92.4%, respectively. At this concentration, flutamide demonstrated an 87.4% reduction in the percentage of viable cells of the PC-3 lineage. The results observed for SLN-BBS-COPA met the criteria established in the ISO:10993-5 standard [57], according to which a material is considered cytotoxic when the percentage of viable cells is less than 70%. SLN at a concentration of 80 µg/mL showed a percentage of viable cells of less than 15%, highlighting the excellent performance of natural bioactives in the cytotoxic activity towards the PC-3 lineage. The percentages of viable cells at 40 µg/mL and 80 µg/mL for SLN-W (without natural bioactives) were 65.3% and 63.61%, respectively. This behavior can probably be attributed to the composition of the SLN, since it is recognized that the composition of the lipid matrix, the type and concentration of the surfactant, and the electrical charge of the surface are also determining factors for the cytotoxicity of SLNs [58,59,60].

The percentage of viable cells in the DU-145 cell line decreased as the concentration of natural bioactives, whether added to SLN or not, increased, similar to what occurred in the PC-3 cell line (Figure 11). Flutamide showed lower percentages of viable cells compared with natural bioactives, both incorporated into SLN (SLN-BBS-COPA, SLN-BBS, SLN-COPA) and not incorporated (BBS, COPA, BBS + COPA). However, BBS + COPA at a concentration of 80 µg/mL exhibited better cytotoxic activity (26.2%) compared with flutamide (42.4%). SLN-BBS-COPA showed a percentage of viable cells of 48% for the DU-145 cell line at 80 µg/mL. Furthermore, at all concentrations, SLN-W showed a percentage of viable cells below 70% (64%, 63%, 58%, and 54% for 10 µg/mL, 30 µg/mL, 40 µg/mL, and 80 µg/mL, respectively). These values are below those recommended by ISO:10993-5 [57]. However, previous studies have shown that non-ionic surfactants, such as Tween 80, may exhibit some degree of cytotoxicity, depending on the surfactant concentration in the formulation [61,62].

However, it is important to note that SLN-BBS showed satisfactory results, with the percentage of viable cells (<70%) at all tested concentrations (10 µg/mL, 30 µg/mL, 40 µg/mL, and 80 µg/mL being 61.9%, 58.3%, 56.2%, and 48.2%, respectively) indicating that BBS also exhibited cytotoxic activity when used alone. On the other hand, this effect was not observed for SLNs containing only COPA (SLN-COPA). The experimental results indicate that the PC-3 and DU-145 cell lines exhibited different sensitivities to the investigated SLNs [58].

## 4. Conclusions

Solid lipid nanoparticles based on babassu oil and copaiba oleoresin were successfully prepared using emulsification–ultrasonication. Previously, the GC-MS spectrum identified lauric acid and β-caryophyllene as chemical markers of BBS and COPA, respectively. It was observed that the hydrodynamic diameter and the polydispersity index of the SLNs were sensitive to the concentrations of stearic acid and Tween 80, as well as to time (0, 7, 15, 30, 40, and 60 days) and temperature (8 °C and 25 °C). This analysis aimed to obtain SLNs with hydrodynamic diameter (HD) and polydispersity index (PDI) values lower than 400 nm and ≤0.3, respectively. SLNs containing BBS and COPA in a 1:1 ratio and kept at 25 °C for 0, 7, 15, 30, 40, and 60 days met all these criteria. Furthermore, TEM images identified a spherical morphology of the SLNs, which is essential during blood circulation and cell interactions. The viability of prostate cancer cell lines PC-3 and DU-145 was investigated when kept in contact with the SLNs developed in this work. The SLN-BBS-COPA formulation showed a percentage of viable cells ≤70% (according to ISO:10993-5) for PC-3 and DU-145 cells at concentrations of 30 µg/mL and 80 µg/mL, respectively. These results indicate that SLNs containing BBS and COPA exhibited satisfactory cytotoxic activity (<70%) towards PC-3 and DU-145 prostate cancer cell lines.

## 5. Patents

The patent associated with this study is under evaluation at the National Institute of Industrial Property (Brazil): Solid lipid nanoparticles (Process number: BR 10 2024 002781 7).

## Figures and Tables

**Table 1:** Decoded values of the independent variables based on the experimental design.

Independent Factors	Minimum Level	Central Point	Maximum Level
Stearic acid	2	4	6
Tween 80	0	2.5	5

**Table 2:** BBS and COPA proportions used for preparing the solid lipid nanoparticles.

SLNs	Natural Bioactive	BBS:COPA Ratio
SLN-BBS-COPA	BBS and COPA	1:1
SLN-BBS	BBS	1:0
SLN-COPA	COPA	0:1
SLN-W	Without natural bioactive	0:0

**Table 3:** Nomenclature, temperature, and time analysis used during the stability study.

SLNs	Temperature (°C)	Time
SLN-BBS-COPA-RT	25 ± 1.0	60 days
SLN-BBS-COPA-UR	8 ± 0.5	
SLN-W-RT	25 ± 1.0	
SLN-W-UR	8 ± 0.5	

**Table 4:** Methyl esters in BBS, identified via CG-MS.

	Analyte (Corresponding Acid)	Retention Time (min)	*m*/*z*
1	Lauric acid	10.230	55/74/87
2	Myristic acid	12.351	55/74/87
3	Palmitic acid	13.703	55/74/87
4	Oleic acid	14.591	55/74/87

**Table 5:** Methyl esters in COPA, identified via CG-MS.

	Analyte (Corresponding Acid)	Retention Time (min)	*m*/*z*
1	α-Copaene	8.435	105/119/161
2	β-Caryophyllene	8.970	69/93/133
3	α-Humulene	9.410	80/93/121

**Table 6:** HD and PDI values measured from SLN compositions recommended via the initial analysis conducted as part of the experimental design.

Samples	Stearic Acid (%)	Tween 80 (%)	HD (nm)	PDI
SLN 1	2	0	242.8 ± 3.20	0.168 ± 0.010
SLN 2	6	0	389.2 ± 64.70	0.522 ± 0.139
SLN 3	2	5	98.0 ± 1.20	0.393 ± 0.026
SLN 4	6	5	259.7± 4.40	0.285 ± 0.008
SLN 5C *	4	2.5	281.0 ± 3.63	0.251 ± 0.049
SLN 6C *	4	2.5	331.9 ± 5.61	0.272 ± 0.026
SLN 7C *	4	2.5	283.9 ± 4.42	0.251 ± 0.032

**Table 7:** The *p*-values, Ftest, and R^2^ obtained from the mathematical adjustment of the linear and quadratic models to the experimental data shown in Table 6.

Response Variables	Mathematical Model	*p*-Value	Ftest	R^2^
HD	Linear	<0.001	60.52	0.919
	Quadratic	<0.001	63.14	0.922
PDI	Linear	0.002	16.72	0.800
	Quadratic	0.012	7.08	0.630

**Table 8:** Concentration of samples used in cell viability of PC-3 and DU-145 cell lines.

SLNs	[] _mean_	[] _BBS_	[] _COPA_
BBS-COPA	10 µg/mL	8.53 µg/mL	11.47 µg/mL
	30 µg/mL	25.58 µg/mL	34.42 µg/mL
	40 µg/mL	34.11 µg/mL	45.89 µg/mL
	80 µg/mL	68.21 µg/mL	91.79 µg/mL

## Data Availability

The research data are available upon demand.
